# The Role of Bitter Taste Receptors in Cancer: A Systematic Review

**DOI:** 10.3390/cancers13235891

**Published:** 2021-11-23

**Authors:** Sofie Zehentner, Agnes T. Reiner, Christoph Grimm, Veronika Somoza

**Affiliations:** 1 Department of Physiological Chemistry, Faculty of Chemistry, University of Vienna, 1090 Vienna, Austria; sofie.zehentner@univie.ac.at (S.Z.); agnes.mistlberger-reiner@univie.ac.at (A.T.R.); 2 Comprehensive Cancer Center Vienna, Gynecologic Cancer Unit, Department of General Gynecology and Gynecologic Oncology, Medical University of Vienna, 1090 Vienna, Austria; christoph.grimm@meduniwien.ac.at; 3 Leibniz Institute of Food Systems Biology at the Technical University of Munich, 85354 Freising, Germany; 4 Chair of Nutritional Systems Biology, School of Life Science, Technical University of Munich, 85354 Freising, Germany

**Keywords:** bitter taste, bitter taste receptors, TAS2Rs, genetic variability, cancer risk, carcinogenesis

## Abstract

**Simple Summary:**

Bitter taste receptors (TAS2Rs) are functionally expressed in various extra-oral cells eliciting non-gustatory functions. In the context of cancer, their functionality is less clear, despite growing interest in recent years. This is the first systematic review that summarizes the current knowledge on the role of TAS2Rs in cancer from different perspectives, from cellular mechanisms of cancer to sensory perception. Although the association between TAS2R-mediated bitterness sensitivity, dietary intake and cancer risk is so far inconclusive, the majority of studies provide evidence for the downregulated gene expression of some functional TAS2Rs in cancerous compared to non-cancerous cell lines and tissue samples. Additionally, the agonist-related activation and overexpression of TAS2Rs per se induced various anti-cancer effects, leading to the hypothesis that TAS2Rs impact carcinogenesis and could serve as a target in cancer therapy by interfering with typical capabilities of cancerous cells, known as the hallmarks of cancer.

**Abstract:**

Background: Since it is known that bitter taste receptors (TAS2Rs) are expressed and functionally active in various extra-oral cells, their genetic variability and functional response initiated by their activation have become of broader interest, including in the context of cancer. Methods: A systematic research was performed in PubMed and Google Scholar to identify relevant publications concerning the role of TAS2Rs in cancer. Results: While the findings on variations of TAS2R genotypes and phenotypes and their association to the risk of developing cancer are still inconclusive, gene expression analyses revealed that TAS2Rs are expressed and some of them are predominately downregulated in cancerous compared to non-cancerous cell lines and tissue samples. Additionally, receptor-specific, agonist-mediated activation induced various anti-cancer effects, such as decreased cell proliferation, migration, and invasion, as well as increased apoptosis. Furthermore, the overexpression of TAS2Rs resulted in a decreased tumour incidence in an in vivo study and TAS2R activation could even enhance the therapeutic effect of chemotherapeutics in vitro. Finally, higher expression levels of TAS2Rs in primary cancerous cells and tissues were associated with an improved prognosis in humans. Conclusion: Since current evidence demonstrates a functional role of TAS2Rs in carcinogenesis, further studies should exploit their potential as (co-)targets of chemotherapeutics.

## 1. Introduction

Cancer patients frequently experience taste alterations, such as bitter taste dysgeusia, as a side effect of chemotherapy [1,2,3], likely by chemotherapeutic drugs interfering with the pathways of cell proliferation of taste cells [4], and even by impacting the expression of taste receptors [5]. As the efficacy of chemotherapeutic treatments was shown to be associated with the recurrence of regular taste perception [6], the risk for bitter taste dysgeusia was hypothesized to be linked to an increasing spread of cancer [7]. This hypothesis is supported by the discovery that altered bitter taste perception even preceded chemotherapy [7,8], and was, therefore, discussed as a side effect of cancer [6]. However, these findings were published in the 1970s and no causal relationship has yet been proven.

Bitter taste perception in humans is mediated by 25 functional bitter taste receptors (TAS2R1, 3, 4, 5, 7, 8, 9, 10, 13, 14, 16, 19, 20, 30, 31, 38, 39, 40, 41, 42, 43, 45, 46, 50, 60) located in the taste cells of the tongue [9]. TAS2Rs belong to the group of G protein-coupled receptors and are activated by a variety of structurally diverse molecules. It is generally agreed that the innate ability to experience oral bitter taste sensation helps to protect from potentially toxic food constituents [9], which often taste bitter [10]. However, TAS2Rs are not only expressed in the oral cavity; they have been also found in the gastrointestinal tract [9,11] and beyond in many extra-oral tissues [11,12,13]. The activation of these ectopically expressed TAS2Rs by bitter-tasting compounds has been associated with tissue-specific, functional responses. The binding of TAS2R agonists triggers a conformational change of these G protein-coupled receptors and consequently activates cellular downstream signalling cascades involving the adenylyl cyclase and/or phospholipase C pathway [11,12,13]. The functional response depends on the cell type which was activated, such as the gustatory sensation of bitter taste [9], a stimulated proton secretion of gastric parietal cells [14], a secretion of satiating hormones such as glucagon-like peptide from entero-endocrine cells [11,12,15], a decreased lipopolysaccharide-mediated interleukin 6 release from gingival fibroblasts [16], an intra-cellular mobilization of calcium leading to the release of nitric oxide and antimicrobial peptides in airway respiratory cells [12,13], and various effects on anti-cancer mechanisms [17,18,19,20,21,22]. The diverse biological functions mediated by TAS2R agonists suggest a potential use of bitter compounds in the prevention and treatment of various diseases [11,12,13,23], including various types of cancer [17,18,19,20,21,22]. TAS2Rs are also present and functionally active in cancerogenic cells [17,18,19,20,21,22,24,25,26]. In in vitro and in vivo experiments, bitter-tasting plant constituents [17,18,20,21,27,28,29,30,31,32] and extracts thereof [33,34] showed anti-cancer activities and/or synergistic effects with chemotherapeutic drugs. Various mechanisms for anti-cancer activities of bitter-tasting plant constituents, some of them involving TAS2Rs, have been demonstrated in these studies [17,18,20,21,27,29,30,31,32,33,34]. However, the functional role of TAS2Rs in carcinogenesis and treatments thereof has not yet been elucidated and is hypothesized in this systematic review by summarizing evidence from the literature.

## 2. Methods

### 2.1. Eligibility Criteria

This systematic review included studies focusing on the role of bitter taste receptors in cancer from different points of view. Case-control and cohort studies, which investigated the possible relationships between gene variations of TAS2R, bitter taste sensitivity, dietary intake, and the risk of developing cancer, were used to summarize the current knowledge on the effects of genetic-mediated bitter taste perception on cancer risk. Studies that examined the gene and protein expression of TAS2Rs, their agonist-mediated activation, the triggered effects at the cellular and molecular levels, and/or effects of survival time of patients were included in the systematic review to gain a better understanding for the role of TAS2Rs in cancer. As the scientific literature about the role of bitter taste receptors in cancer is still an emerging field and only a very limited number of studies on this topic are available, all articles meeting the search strategy were included to provide a comprehensive overview on the current state of knowledge and encourage further research.

### 2.2. Search Strategy and Study Selection

The systematic research was carried out using two electronic databases, namely Google Scholar and PubMed. The search terms “bitter taste”, “bitter taste sensitivity”, “bitter taste receptors” or “TAS2R” or “T2R” and “TAS2R variations” or “TAS2R polymorphisms” were combined with “cancer”, “carcinoma”, “cancer cell or tissue” and “cancer risk” to give a comprehensive overview of the literature. If the titles of the proposed studies indicated that they had investigated at least one of the three main parts of these systematic review, then the abstract was additionally screened. Afterwards, the full texts of the relevant articles were reviewed, and the reference was imported to Endnote X9 (Thomson Reuters, New York, NY, USA). Furthermore, the reference list of these publications was examined for further suitable studies. Additionally, the Food Systems Biology Database [35] was used to identify further TAS2Rs of the investigated agonists and vice versa. The most recent literature search was carried out in June 2021. This systematic review was registered on the Open Science platform and can be found via the registration identifier osf-registration-wsj2q-v1.

## 3. Results

### 3.1. Associations between Genetic Variability of Bitter Taste Receptors and Cancer Risk

Several recent studies [36,37,38,39,40,41,42,43,44,45,46] have examined the role of bitter taste perception in cancer development, focusing on the question of whether genetic variations of *TAS2Rs* impact the risk for developing cancer directly as well as indirectly via different dietary intake caused by the gene-mediated variability of bitter taste sensitivity. Within the scientific community, the following hypotheses from different perspectives are discussed: (i) an increased bitterness perception leads to the avoidance of bitter-tasting foods with health-promoting phytochemicals and, as a consequence, increases cancer risk [36,38]; (ii) an elevated sensitivity of TAS2Rs to bitter-tasting compounds is protective against bitter-tasting carcinogenic compounds, leading to a decreased cancer risk [37,40,41]; (iii) cancer risk is influenced by genetic variants via TAS2R-related, tissue-specific functions [44].

Indeed, an association between an elevated risk for cancer development and the genotypes of various bitter taste receptors was identified [37,40,41,43,44,45], although this association was not always transferable to the actual dietary intake [36,37,39,45]. So far, it is not conclusive which of the bitter taste receptor genotypes might pose a higher risk of carcinogenesis.

Among the limited numbers of studies [36,37,38,39,40,41,42,43,44,45,46] that examined possible direct or indirect relationships between polymorphisms of *TAS2Rs*, bitter taste sensitivity and the risk for developing cancer, only a few studies included sensory evaluations of bitter taste perception [36,38] and dietary intake [36,37,38,39,45], whereas genotyping analyses of *TAS2R*s were performed in most investigations [36,37,39,40,41,42,43,44,45,46]. In the latter studies, the *TAS2R38* gene was of special interest, because its polymorphisms are associated with the sensitivity to the bitter-tasting compounds propylthiouracil (PROP) and phenylthiocarbamide (PTC), enabling the evaluation of bitter taste and division into different phenotypes [47]. While the PAV/PAV diplotype is linked to the highest sensitivity to PROP and PTC, the heterozygous PAV/AVI diplotype leads to an intermediate sensitivity. Therefore, these variants elicit tasting phenotypes. Since carrying the AVI/AVI diplotype results in the lowest sensitivity, its carriers are so-called non-tasters. Apart from PROP and PTC, the bitter taste sensitivity to naturally occurring compounds such as goitrin and sinigrin is affected by the *TAS2R38* diplotype as well [47]. Therefore, studying the genetic variations of the *TAS2R38* is a promising approach to identify possible associations between bitterness perception and susceptibility to cancer [36,37,38,39,40,41,45,46], although the knowledge regarding cancer type specificity is limited.

Most studies investigating potential relationships between the genetic variability of *TAS2R*s, bitter taste sensitivity, dietary intake, and the risk of developing cancer focused on tumours along the gastrointestinal tract [37,38,39,40,41,42,43,45,46]. Since these tissues are directly exposed to bitter-tasting food constituents, this focus appears to be obvious. First, along the digestive tract, oral health might be affected by bitter compounds. However, no case–control study which investigated the potential relationship between genetic variations within TAS2Rs, bitter taste sensitivity, dietary intake, and susceptibility to oral cancer could be found. Therefore, starting with the stomach, a case–control study in a Korean population [37] showed an increased gastric cancer risk (OR—1.513; 95% CI—(1.148, 1.994); *p* = 0.001) associated with the PAV/AVI diplotype of *TAS2R38*, which evokes an intermediate bitter taste sensitivity to PROP and PTC. However, food consumption was not associated with different *TAS2R38* diplotypes, suggesting a diet-independent effect of gene variations on gastric cancer risk [37]. On the contrary, another genotyping study [40] revealed that the AVI/AVI diplotype occurred more frequently among Japanese patients with gastrointestinal cancer compared to controls, while the PAV/PAV diplotype occurred less frequently. Therefore, non-tasters were more susceptible to gastrointestinal cancer (OR—2.04; 95% CI—(1.095, 3.815); *p* = 0.024), whereas carrying the sensitive PAV/PAV diplotype seemed to be protective (OR—0.55; 95% CI—(0.299, 0.996); *p* = 0.048). No significant differences in genotype frequencies were detected for *TAS2R46* polymorphisms in this case–control study [40].

Sensory evaluations, carried out by Basson et al. [38], showed that the bitterness perception of PROP, targeting TAS2R38 [35,48], was weakly positively correlated (r = 0.24; *p* < 0.01) with the number of colonic neoplastic polyps in men older than 66 years, but not in younger men. The perception of the bitter taste of PROP and age were inversely associated with vegetable consumption, but, contrary to expectations, vegetable consumption per se did not indicate a higher incidence of polyp number, which is a known risk factor for colon cancer [38]. In contrast, in a multi-ethnic case–control study [39], neither the risk for developing colorectal adenoma nor most selected dietary variables were associated with several genetic variations of the bitter taste receptor genes *TAS2R16*, *TAS2R38* and *TAS2R50*. Only marginal negative associations could be observed between the intake of dietary fibre, vegetables and non-starchy vegetables and the investigated polymorphism of *TAS2R50* [39].

Effects on the dietary intake due to genetic variations within the *TAS2R38* gene could also not be found in a case–control study in an Asian population, but carrying the AVI/AVI diplotype was associated with a lower risk of colorectal cancer (OR—0.74; 95% CI—(0.56, 0.99); *p* = 0.021) [45]. In contrast to this study, in a case–control study on Caucasians [41], a higher risk for colorectal cancer (OR—1.34; 95% CI—(1.12, 1.61); *p* = 0.001) was found for non-tasters by comparing all the diplotypes of *TAS2R38* linked to non-tasting phenotypes, with the diplotypes eliciting tasting phenotypes. No significant associations between single-nucleotide polymorphisms (SNPs) of the *TAS2R38* gene and colorectal cancer risk were detected [41]. A meta-analysis [46] of the studies detailly described herein [37,39,40,41,45] could not find any associations between the common genetic variations within the *TAS2R38* gene and the risk for neoplasm along the gastrointestinal tract [46]. Similarly, no associations were found for neither SNPs within the *TAS2R14* gene [42] nor within *TAS2R16* [43] and colorectal cancer risk. However, one of the six investigated SNPs in *TAS2R16* showed a weakly positive association with the risk for developing rectal cancer (OR—1.62; 95% CI—(1.06, 2.47); *p* = 0.03) [43].

Apart from cancer types along the gastrointestinal tract, only the following three studies examined a potential relationship between bitter taste sensitivity, bitter taste receptors and cancer incidence of malignant cancers in general [36], pancreatic cancer [49], and papillary thyroid carcinoma [44]. In the UK Women’s Cohort Study [36], an elevated risk for malignant cancers was observed for tasters (HR—1.40; 95% CI—(1.03, 1.90); *p* = 0.030) and supertasters (HR—1.58; 95% CI—(1.06, 2.36); *p* = 0.024) among women older than 60 years, whereas younger supertasters (HR—0.54; 95% CI—(0.31, 0.94); *p* = 0.031) had a decreased risk compared to non-tasters. Interestingly, these results were not reflected by analysing cancer incidence in relation to the diplotypes of *TAS2R38*. The classification into different types of tasters was based on the perceived magnitude of the bitter taste of PTC in sensory tests. While tasters experienced an intermediated bitter taste of PTC and PROP, supertasters described it as extremely bitter, whereas non-tasters did not perceive its bitter taste [36]. In this approach of classification, it has to be considered that the perceived bitterness of PTC and PROP is partially influenced by other factors beyond the *TAS2R38* genotype [47,50], such as the level of mRNA expression of PAV-*TAS2R38* [51] and the number of fungiform papillae [50,52], which is impacted by an SNP within the gustin gene encoding for the taste bud trophic factor gustin [52].

Concerning the risk for pancreatic cancer, no association between any SNPs in *TAS2Rs* was detected with this type of cancer by analysing genotyping datasets from different genome-wide association studies [49].

In a case–control study [44], a combined appearance of two SNPs within *TAS2R3* and *TAS2R4* was associated with a decreased risk of papillary thyroid carcinoma (OR—0.59; 95% CI—(0.36, 0.97); *p* = 0.036) and linked with lower total triiodothyronine concentration in the serum, indicating that genetic variations of *TAS2Rs* can impact tissue-specific functions, which might play a role in carcinogenesis. Other genetic variations in five *TAS2R*s did not have a direct impact on this type of cancer nor on any of the analysed thyroid markers [44].

In summary, the hypothesis that bitter taste sensitivity affects the intake of bitter-tasting foods and, as a consequence thereof, impacts the risk for developing cancer, could not be confirmed [36,37,39,45], further leading to the theory that genetic variations of bitter taste receptors could modify the risk of cancer development directly. However, it is not conclusively evident yet which kind of genetic variability might be protective or cancer-promoting. On the one hand, an elevated prevalence for neoplastic polyps, a cancer risk factor [38], and malignant tumours [36] was associated with tasting phenotypes, assessed by sensory evaluation, in older patients. On the other hand, the related tasting *TAS2R38* genotypes and diplotypes were predominately linked to a lower cancer risk, suggesting a protective effect against bitter-tasting carcinogenic compounds for gastrointestinal types of cancer [37,40,41]. Since other studies did not find any evidence for associations between the genetic variations of different *TASR2s* and the susceptibility to these types of cancer [39,42,43,46,49] or even identified a decreased risk for the non-tasting *TAS2R38* diplotype [45], the question of whether specific TAS2Rs and/or their variants, resulting in a distinct bitter sensitivity, play a functional role in carcinogenesis in a population group characterized by dietary habits and/or socio-demographic factors such as age or sex has still to be elucidated.

To better understand the role of bitter taste sensitivity in cancer risk, more sensory evaluations need to be performed to investigate the effects of genetic variations on phenotypes regarding the variability of bitter taste sensitivity as well as individual expression levels. Furthermore, genetic variabilities of *TAS2R*s other than *TAS2R38* should be taken into consideration since they might affect bitter taste sensitivity and dietary intake as well. Moreover, more detailed sensory evaluations, not only including taste threshold levels, but also more detailed sensory descriptors, i.e., time intensity profiles of the bitterness perceived from individual compounds ingested with a complex food matrix, as well as quantitative analysis of the dietary intake would be meaningful to identify yet unknown relationships. Since TAS2Rs are not only present in taste cells, but also in extra-oral tissues [9,11,12,13], including cancer cells and tissues [17,18,19,20,21,22,24,25,26], the different genetic variants could also affect cancer-associated functions of TAS2Rs.

### 3.2. Gene and Protein Expression of Bitter Taste Receptors in Cancer

The analysis of expression levels of bitter receptors in cancerous and non-cancerous cells contributes to a more profound understanding of the relevance of TAS2Rs in cancer biology as well as treatment. Therefore, several studies investigated the gene expression of *TAS2Rs* in cancerous and non-cancerous cell lines and tissues [17,18,19,20,21,22,24,25,26]. While most studies focusing on the susceptibility to cancer analysed cancer types along the gastrointestinal tract, the expression of bitter taste receptors at the gene and protein levels and their regulation were only analysed in the context of non-gastrointestinal tumours [17,18,19,20,21,22,24,25,26]. Moreover, most of these studies tested whether bitter-tasting compounds could activate the expressed bitter taste receptors in cancer cells and might bear potential in cancer therapy [17,18,19,20,21,22,24,26]. To better distinguish between the results of gene and protein expression of bitter receptors, we followed the nomenclature guidelines recommended by the Gene Nomenclature Committee of the Human Genome Organization [53]. Consequently, bitter receptor genes are named as *TAS2Rx* and the related protein in non-italic font as TAS2Rx.

Singh et al. [24] reported a lower mRNA expression level of *TAS2R4* in two breast cancer cell lines (MCF-7 and MDA-MB-231) compared to a non-cancerous mammary epithelial cell line (MCF-10A). Other bitter taste receptors (*TAS2R1*, *TAS2R10*, *TAS2R20* (*49*) and *TAS2R38*) showed a generally low expression level in comparison to breast cancer markers and no differences among the three cell lines. Similar results were obtained for the protein expression of TAS2Rs on the cell surface by flow cytometry analysis. Stimulation with various bitter agonists showed a concentration-dependent effect on calcium mobilization in all cell lines, with the highest response in non-cancerous cells (MCF-10A), and thereby confirmed the functional activity of TAS2Rs [24]. Although the results of the calcium mobilization assay are in line with the results of expression analyses, it is highly recommended to normalize data to reference genes equally expressed in all cell lines. Conversely, an nCounter analysis of the mRNA expression levels of all *TAS2Rs* in the metastatic breast cancer cell line (MDA-MB-231) and normal breast cell line (MCF-10A) revealed a higher relative expression of the two predominantly expressed *TAS2Rs*, namely *TAS2R14* and *TAS2R20*, in the cancer cell line, whereas no differences were detected for other *TAS2Rs* [25]. Further analyses of the intracellular calcium mobilization during exposure to the bitter-tasting compounds quinine or apigenin in combination with sh-RNA knockdown experiments proved that TAS2R4 and TAS2R14 can be activated with agonists in MDA-MB-231 and MCF-10A cells [17]. Investigations of breast cancer and normal breast tissues reinforced the fact that the expression pattern of bitter taste receptors was changed under this pathological state: RT-qPCR-analysis demonstrated *TAS2R4* to be significantly lower and *TAS2R14* significantly higher expressed in breast cancer tissues compared to non-cancerous breast tissues. These results were confirmed at the protein level by Western blot analysis, although statistical significance was only reached for TAS2R14 and not for TAS2R4, possibly due to a limited number of samples from normal and cancerous breast tissues for Western blot analyses [17].

Apart from studies on breast cancer, the mRNA expression levels of some *TAS2Rs* were investigated in several ovarian and prostate cancer cell lines, ovarian cancer tissue and one uterine adenocarcinoma cell line (HEC-1a) [18]. The RT-qPCR analysis showed that *TAS2R14* was downregulated in most cancer cell lines and cancer tissue. In more detail, the downregulated gene expression of *TAS2R1*, *4*, *10*, and *14* varied in ovarian cancer cell lines of different histotypes and ovarian cystadenocarcinoma tissue in comparison to tissue samples from which these cancer cells are supposed to originate. Interestingly, the expression of *TAS2R38* was almost entirely below the limit of detection in all studied epithelial ovarian cancer cell lines, the ovarian cancer tissue and in HEC-1a. In prostate cancer cell lines, *TAS2R38* was significantly attenuated in two of three cell lines, while the expression pattern of the other investigated *TAS2Rs* was always diminished in relation to the expression level in a benign prostatic hyperplasia cell line (BPH1) [18].

Stern et al. [20] reported the mRNA levels of *TAS2R10* in eight human pancreatic cancer cell lines as well as in 75% of primary tissue of human pancreatic ductal adenocarcinoma samples. By means of immunohistochemical analysis, the related TAS2R10 protein was detectable in 79% of another cohort of pancreatic tumour tissue samples and the protein was located intracellularly as well as on the cell surface [20]. Similarly, through a histological analysis of pancreatic ductal adenocarcinoma tissues, TAS2R38 was detected in 78% of the tissue samples, predominantly in cytoplasm and additionally associated with liposomes. TAS2R38 was found in tumour-infiltrating leukocytes as well, but not in non-cancerous pancreatic cells of the tissue samples. The functionality of TAS2R38 and its co-location with lipid droplets was confirmed in a pancreatic cancer cell line and a pancreatic stellate cell line (RLT) [26].

A study [22] examining the expression of *TAS2R8* and *TAS2R10* in human neuroblastoma cells described a lower expression level of both genes in a less differentiated and more malignant cell line (SK-N-BE(2)C) in comparison to a more differentiated and less malignant cell line (SH-SY5Y). Moreover, the functional activity of both bitter taste receptors could be induced by denatonium benzoate [22], a bitter-tasting agonist of TAS2R4, 8, 10, 13, 39, 43, 46, 47 [35,48].

In primary acute myeloid leukemic cells and in human leukaemia cell lines (OCI-AML3 and THP-1), all 25 *TAS2Rs* were identified. The exposure of both primary cells and cell lines to quinine, a bitter-tasting agonist of TAS2R4, 7, 10, 14, 39, 40, 43, 44, 46 [35,48] or denatonium benzoate increased cytosolic calcium concentrations, indicating that TAS2Rs are functionally active [19].

In a recently published study [21], the gene expression of all 25 TAS2Rs was analysed in various cell lines of head and neck squamous cell carcinoma (SCC4, SCC15, SCC47, SCC90, SCC152, OCTT2 and VU147T) as well as in cancer tissues form oral cavity or oropharynx cancer patients (*n* = 10), including tissues obtained from the contralateral, non-cancerous site. In total, no differences of the expression level between the cancerous and non-cancerous tissues could be detected, but additional analyses revealed some up- and downregulations at the individual patient level. Variably expressed *TAS2Rs*, including high levels of *TAS2R4*, *14*, *19*, *20*, *30*, *43* and *45*, were detected in the investigated head and neck squamous cell carcinoma cell lines. By means of immunofluorescence microscopy, TAS2R42 and TAS2R13 were partially localized to the nucleus in SCC47 and SCC4, while TAS2R4, 8, 10, 14, 30 and 46 were detected intracellularly and at the cell membrane in the same two cell lines. Different TAS2R agonists including the multi-targeting agonists denatonium benzoate and quinine led to cytoplasmic and especially to higher nuclear calcium release, proving the functionality of TAS2Rs in various head and neck squamous cell carcinoma cell lines [21].

The results of these studies [17,18,19,20,21,22,24,25,26] are summarized in Table 1. This overview highlights that TAS2Rs are expressed in cancer cells and can be activated by respective agonists. Furthermore, if differences of the gene expression level were detected, a reduced gene expression of *TAS2Rs* in cancerous compared to non-cancerous cell lines and tissues was observed in most cases [17,18,22,24], while only a few upregulations were found [17,25]. TAS2R wide protein expression analyses are limited by the availability of verified antibodies, and are only available for a very limited subset of TAS2Rs. However, it seems likely that inter-individual variabilities exist among cancerous tissues and cells [17,19,20,21], which have not been standardized for influencing factors, like cell phase and differentiation state. Therefore, it is questionable if results from just one or a few non-cancerous tissue samples sufficiently represent the expression pattern under non-cancerous state and, therefore, are suitable to detect general dysregulation. Consequently, non-cancerous tissues should be analysed to a representative extent to know the expression level and variability under healthy conditions or at least in the surrounding, non-cancerous tissue of a cancer. Furthermore, since different expression patterns of TAS2Rs were also observed in cancer cell lines of the same cancer type [18,19,20,21,24,26], the results from several cell lines should be compared for reliable conclusions. Additionally, the gene and protein expressions of all TAS2Rs need to be examined in order to identify the receptors which are relevant in the context of cancer, because so far studies mostly focused on a few of the 25 TAS2Rs.

### 3.3. Functional Role of Bitter Taste Receptors in Cancer Cells and Tissues

Next to the expression patterns of bitter taste receptors in cancer cells and tissues, their functional role is of the utmost interest in the context of cancer biology and treatment. The effects of diverse bitter substances on cellular functions in cancer cells have been analysed in several studies [17,18,19,20,21,22,26] that are discussed in the following paragraphs.

The activation of TAS2R4 and TAS2R14 by quinine or apigenin led to a significant concentration-dependent reduction in cell proliferation of the breast cancer cell line MDA-MB-231, while proliferation of MCF-10A, the normal breast epithelial cells, was only attenuated at higher ligand concentrations [17]. In addition to the anti-proliferative response, the activation of TAS2R4 and TAS2R14 was linked to pro-apoptotic as well as anti-migratory effects in MDA-MB-231. The receptor specificity of these effects was proven by sh-RNA knockdown experiments [17].

In the epithelial ovarian cancer cell line SKOV3, exposure to noscapine, a TAS2R14 agonist [35,48], led to increased pro-apoptotic and decreased pro-survival protein expression [18]. The association between TAS2R14 activation and apoptotic processes was confirmed by knockdown experiments using TAS2R14-targeted siRNA. In more detail, the number of AnnexinV-positive cells, a marker for apoptosis, significantly increased by 17% after noscapine treatment, while no effect was demonstrated for TAS2R14 knockdown SKOV3. A decrease in cell viability through the activation of TAS2R14 was not only shown for noscapine, but also for diphenhydramine [18], another TAS2R14 agonist [35,48]. Furthermore, the overexpression of TAS2R14 enhanced these effects. Similarly, the activation of TAS2R14 reduced cell survival in prostate cancer cell lines too [18].

The activation of the bitter taste receptor TAS2R10 by caffeine in pancreatic cancer cell lines affected different signalling pathway molecules including the downregulation of ABCG2, a protein promoting multidrug resistance. The specific role of TAS2R10 was proven by siRNA knockdown [20]. Controversially, the stimulation of TAS2R38 by PTC, as specific agonist of TAS2R38 [35,48], or AHL-12, a bacterial molecule, induced the upregulation of the protein ABCB1, which fosters multidrug resistance in a pancreatic cancer cell line [26]. There is significant evidence that cytotoxic effects of gemcitabine or 5-fluorouracil on pancreatic tumour cell lines are synergistically enhanced by the bitter-tasting food constituent caffeine through at least a partial activation of TAS2R10 [20]. However, other TAS2Rs which are known to be activated through caffeine, such as TAS2R7, 14, 43 and 46 [35,48], were not investigated. Synergistic effects of caffeine with the cytotoxic effect of cisplatin have been reported in hepatocellular carcinoma cells before [28], but without investigating the role of bitter taste receptors by, e.g., siRNA knockdown experiments. This is of importance, since patients with TAS2R10-expressing pancreatic tumours had a chance of prolonged survival compared to TAS2R10 negative controls [20], whereas no correlation was seen between the expression frequency of TAS2R38 and survival time in another study involving pancreatic cancer patients [26].

Anti-cancer effects were also observed to be induced by TAS2R activation in acute myeloid leukaemia cells [19]. The in vitro exposure of these cells to the TAS2R agonist denatonium benzoate resulted in alterations of the mRNA levels of genes involved in cellular processes associated with cancer, such as apoptosis, cell cycle, DNA damage, migration, and metabolism. Moreover, the activation of TAS2R targeted by denatonium benzoate, such as TAS2R4, 8, 10, 13, 39, 43, 46, 47 [35,48], negatively impacted cancer cell physiology, motility, and cell proliferation, while the latter was caused by enhanced cell cycle arrest and apoptosis. Additionally, the in silico gene expression profiling of acute myeloid leukaemia cell samples from a database revealed that lower expression levels of *TAS2R5*, *TAS2R9*, *TAS2R10* or *TAS2R14* were linked with some clinical parameters, i.e., *TP53* and *TET2* mutations, as well as higher molecular and cytogenetic risks, indicating poor prognosis [19].

In a recently published study [21], the nuclear calcium response triggered by TAS2R agonists decreased metabolic activity and led to mitochondrial dysfunctions, further resulting in increased caspase activity and therefore in apoptosis in head and neck squamous cell carcinoma cell lines. Additionally, higher mRNA level of *TAS2Rs* in total and in particular of *TAS2R4* in tissue samples from head and neck squamous cell carcinoma were linked to a higher survival rate, supporting the in vitro results concerning the impact of TAS2Rs on cancer cell proliferation [21].

A study on the functionality of TAS2Rs in neuroblastoma [22] showed an inhibition of the self-renewal capacity as well as migration and invasion, due to over-expression of TAS2R8 or TAS2R10 in transfected SK-N-BE(2)C, which is a less differentiated neuroblastoma cell line. In more detail, a reduced gene expression and enzymatic activity of matrix metalloproteinase-2 (MMP-2), and therefore suppressed invasion and migration abilities were observed in TAS2R8 or TAS2R10 upregulated SK-N-BE(2)C cells. Additionally, the hypoxia-induced gene expression of hypoxia-inducible factor-1α (*HIF-1α*) and its mediated genes, vascular endothelial growth factor (*VEGF*) and glucose transporter-1 (*GLUT1*), were downregulated too, suggesting a possible counteracting effect on metastasis, angiogenesis, and glucose metabolism. Further in vitro experiments highlighted that the overexpression of both receptors induced differentiation processes and suppressed the expression of cancer stem cell markers in this cell line. Moreover, in vivo experiments with mice showed that the subcutaneous injection of SK-N-BE(2)C cells, overexpressing *TAS2R8* or *TAS2R10* by stable transfection, reduced tumour incidence by 80% and 30%, respectively. Furthermore, a trend towards reduced tumour growth compared to the xenograft control group was shown. The upregulation of *TAS2R8* and *TAS2R10* counteracted gene expression of *MMP-2* and *P-selectin* in tumour tissues, indicating an improved cell adhesion and therefore a reduced metastatic potential [22].

**Figure 1:**
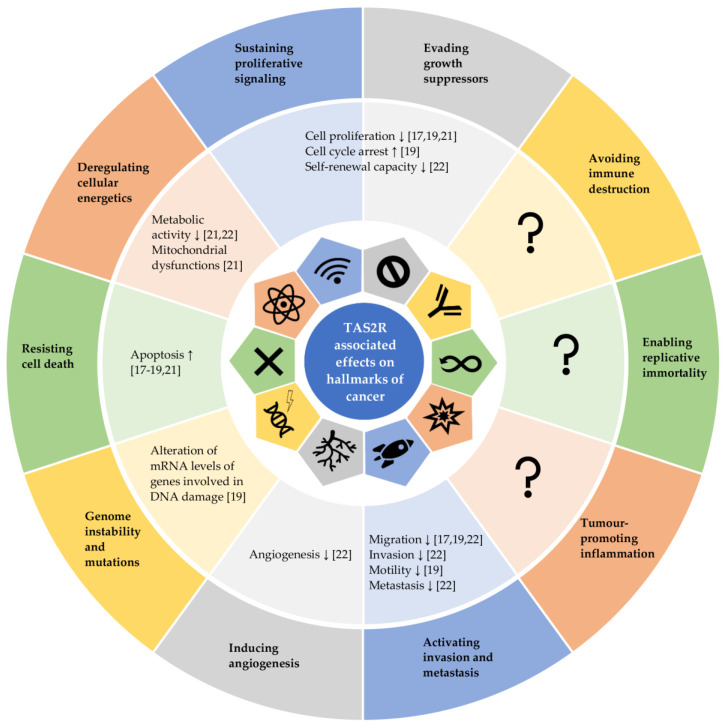
This figure is adapted from Hanahan and Weinberg, 2011 [54] and highlights how agonist-mediated activation of TAS2Rs and/or by overexpression of TAS2Rs can counteract the diverse capabilities of cancerous cells and tissues, known as hallmarks of cancer. ↑: promoting effect; ↓: inhibitory effect.

In summary, the agonist-mediated activation [17,18,19,20,21] and even just overexpression [22] of the analysed TAS2Rs are predominantly associated with anti-cancer effects. In more detail, TAS2Rs are suggested to play a relevant role in cell biology of various cancer types [17,18,19,20,21,22,26]. Their activations are linked with decreased cell survival [17,18,19,20,21], supporting the hypothesis that downregulation of TAS2Rs by cancerous cells might help to avoid growth suppression by the microenvironment [19,24] while a higher expression of TAS2Rs could improve survival time of cancer patients [20,21]. Additionally, there is evidence that TAS2Rs act as regulator by suppressing cancer stem cell markers and other typical traits of cancer cells such as angiogenesis, invasion, metastasis [22], migration [17,19,22], and metabolic activity [21,22]. As the activation of bitter taste receptors such as TAS2R50 counteracts inflammatory stimuli [16], it should be investigated, whether anti-cancer effects triggered by TAS2Rs activation and higher expression of *TAS2Rs* interfere with tumour promoting inflammation processes, resulting in further anti-cancer effects. Figure 1 highlights how the agonist-mediated activation of TAS2Rs and/or the overexpression of *TAS2Rs* can counteract the diverse capabilities of cancerous cells and tissues, known as the hallmarks of cancer.

Although several TAS2Rs-related mechanisms have already been identified [17,18,19,20,21,22,26], the TAS2R-mediated downstream signalling pathway still needs to be further investigated. Some TAS2Rs, such as TAS2R10, 14 or 46, are known to be activated by a large number of structurally divers bitter-tasting compounds, while TAS2R1, 4, 7, 39, 40, 43, 44 and 47 are activated by a more limited set, and others, mainly TAS2R3, 5, 8, 13, 20 or 50, are specialized to interact with just a few [48]. Furthermore, TAS2R16 and 38 are restricted to specific chemical motifs that are present in several bitter compounds [48]. Consequently, the diverse structural spectrum of bitter compounds acting as agonists of specific TAS2Rs has to be taken into consideration. Reciprocally, some bitter compounds are able to bind to multiple TAS2Rs. In this context, effects on anti-cancer mechanisms have been reported for several bitter-tasting compounds, but without analysing the potential involvement of bitter taste receptors as targets. Some examples of such bitter compounds are: cucurbitacin B [27], an agonist for TAS2R10 and TAS2R14 [35], parthenolide [29], an agonist for TAS2R1, 4, 8, 10, 14, 44, 46 [35], caffeine [28,30], an agonist for TAS2R7, 10, 14, 43, 46 [35] and noscapine [31,32], an agonist for TAS2R14 [35]. More information regarding TAS2R binding patterns and agonists can be retrieved from the Food Systems Biology Database [35].

These promising research results suggest TAS2Rs as potential target of cancer therapy [17,18,19,20,21,22] and additionally emphasize an involving function of bitter taste receptors in carcinogenesis. In this context, potential interactions of chemotherapeutic drugs with TAS2Rs arouse interest. So far, taste qualities of chemotherapeutic drugs are largely unknown. However, chemotherapeutic drugs might induce taste perception after passing into saliva and through the venous taste phenomenon [55]. This potential mechanism could shed light on the mechanisms for chemotherapy-induced dysgeusia, especially for bitter and metallic taste, further highlighting that chemotherapeutic drugs could activate TAS2Rs in cancer cells too, additionally boosting the anti-cancer effects of the chemotherapeutic drugs.

## 4. Conclusions and Outlook

So far, there is no conclusive evidence that cancer risk depends on the dietary intake of bitter-tasting food constituents mediated by genetic-related bitter taste sensitivity [36,37,39,45]. It seems more likely that TAS2Rs impact cancer etiology via distinct activation and genetic variations per se. However, the underlying pathways and mechanisms affecting the risk of cancer development through *TAS2R* genotypes still need to be investigated. Studies elucidating the impact of genetic variations on physiological function of TAS2Rs along the gastrointestinal tract and beyond are mandatory to identify and understand the involvement of TAS2Rs in the susceptibility to and progression of cancer at the molecular level and additionally could contribute to a better understanding of the potential role of TAS2Rs in cancer aetiology and therapy. The ectopic expression and activation of TAS2Rs in various cancer cells and tumour tissues have already been proven [17,18,19,20,21,22,24,25,26], as well as the functionality by the use of several agonists [17,18,19,20,21,22,24,26]. Both responses, down- and upregulations, were reported among TAS2Rs [17,18,21,22,24,25], though downregulations of various TAS2Rs, were more prevalent [17,18,22,24]. Regardless of the kind of dysregulation, the underlying mechanisms and the effect on cancer progression are poorly explored. Nevertheless, the detected differential expression levels of *TAS2Rs* [17,18,21,22,24,25], mostly reported for *TAS2R1, 4*, 8, *10*, *14* and *38* so far, underline their potential functional role in carcinogenesis.

As highlighted in this article, recent studies showed that the activation of bitter taste receptors in cancer cells was predominantly associated with anti-cancer effects [17,18,19,20,21] and the downregulation of some *TAS2Rs*, i.e., *TAS2R4*, *5*, *9*, *10* or *14*, was associated with poor prognosis [19,20,21], while overexpression counteracted cancer-promoting effects [22]. These results also support the hypothesis that TAS2Rs play a functional role in carcinogenesis. In more detail, we hypothesize that their activation counteracts most typical capabilities of cancerous cells during cancer development and progression, known as hallmarks of cancer, such as avoiding growth suppression, deregulating cellular energetics and apoptosis as well as promoting invading and metastatic capabilities. This conclusion is in line with the findings of *TAS2R* downregulation in cancer.

In this context, knowledge regarding the impact of chemotherapeutic drugs, in particular those possibly acting as ligands of TAS2Rs, on TAS2R-mediated expression and function, such as bitter perception or cell proliferation and apoptosis, is also needed. If TAS2R expression is specifically altered, not only in taste cells, potentially leading to dysgeusia, but also in cancer cells, the hypothesis of bitter taste dysgeusia becoming an indicator of chemotherapeutic efficacy should be explored. Furthermore, if effects of cytostatic drugs are mediated through the activation of TAS2Rs, the interindividual gene variations of *TAS2Rs* resulting in variability of bitter taste sensitivity might impact treatment efficacy. Therefore, genetic variations might need to be taken into consideration when decisions on treatments are made.

In general, further research is required to achieve a better understanding of the functional role of ectopically expressed bitter taste receptors in cancerous cells and their potential as chemotherapeutic targets to provide promising novel strategies for cancer treatment and to improve chemotherapy while reducing side effects.

## Figures and Tables

**Table 1:** Overview of expression level of bitter taste receptors at gene and protein level in cancerous cell lines and tissues compared to non-cancerous cell lines and tissues as well as analysed agonists.

Cancer Type	Cells or Tissues	*TAS2Rs*/TAS2Rs Regulation	Agonists Used in Functional Assay with Cell Lines	Ref.
Breast cancer	MCF-7 and MDA-MB-231 vs. MCF-10A	*TAS2R4* ↓, TAS2R4 ↓ No differences for TAS2R1, 10, 20 (49), 38 at gene and protein level	Quinine, dextromethorphan, PTC (verified with MCF-7, MDA-MB-231, MCF-10A)	[24]
Breast cancer	MDA-MB-231 vs. MCF-10A	*TAS2R14* ↑, *TAS2R20* ↑ No differences for other *TAS2Rs*		[25]
Breast cancer	Breast cancer tissues vs. non-cancerous breast tissues	*TAS2R4* ↓ *TAS2R14* ↑ TAS2R14 ↑	Quinine, apigenin (verified with MDA-MB-231 andMCF-10A)	[17]
Ovarian cancer	OVCAR4, OVCAR8, ovarian cystadenocarcinoma tissue vs. normal fallopian tube tissue; SKOV3 and IGROV1 vs. normal uterine tissue	*TAS2R1* ↓ in OVCAR8, *TAS2R4* and *14* ↓ in OVCAR8 and SKOV3, *TAS2R10* and *14* ↓ in cancer tissue, *TAS2R38* ↓ in all analysed cell lines and cancer tissue	Noscapine and diphenhydramine for TAS2R14 (verified with SKOV3)	[18]
Endometrial cancer	HEC-1a vs. normal uterine tissue	*TAS2R1, 14, 38* ↓		[18]
Prostate cancer	PC3, LNCAP, DU145 vs. BPH1	*TAS2R1, 4, 10, 14* ↓ in PC3, LNCAP, DU145, *TAS2R38* ↓ in LNCAP, DU145	Noscapine for TAS2R14 (verified with PC3, DU145)	[18]
Pancreatic cancer	Primary human pancreatic tumour tissues, T3M4, SU.86.86, PANC-1, MiaPaCa-2, Colo357, Capan-1, BxPC-3, AsPC-1	*TAS2R10* TAS2R10	Caffeine (verified with BxPC-3 and PANC-1)	[20]
Pancreatic cancer	Tumour infiltrating leukocytes; pancreatic tumour tissues vs. non-cancerous pancreatic tissues; SU.86.86, T3M4, MiaPaCa-2, and RLT	TAS2R38	PTC, N-acetyl-dodecanoyl-homoserine lactone (AHL-12) (verified with SU.86.86 and RLT)	[26]
Neuroblastoma	SK-N-BE(2)C vs. SH-SY5Y	*TAS2R8* ↓ *TAS2R10* ↓	Denatonium benzoate (verified with SK-N-BE(2)C)	[22]
Acute myeloid leukemia	Primary acute myeloid leukemia cells, in silico gene expression profiling of samples from a database, OCI-AML3, THP-1	Analyses of 18 to 24 *TAS2Rs* out of 25 *TAS2Rs* & identification of all *TAS2Rs* but not in all approaches	Denatonium benzoate, quinine (verified with primary acute myeloidleukemia cells, OCI-AML3, THP-1)	[19]
Head and neck squamous cell carcinoma	Cancerous vs. non-cancerous tissues; SCC4, SCC15, SCC47, SCC90, SCC152,OCTT2 and VU147T	Analysis of all *TAS2Rs,* highest gene expression of *TAS2R4*, *14*, *19*, *20*, *30*, *43*, *45* in cancer cell lines, TAS2R4, 8, 10, 13, 14, 30, 42, 46 in SCC4, SCC47, TAS2R4, 8, 10, 30, 39 in SCC47, SCC90, SCC152	Denatonium benzoate, quinine, thujone, diphenidol, flufenamic acid, parthenolide, N-3-oxo-dodecanoyl-L-homoserine lactone (3-oxo-C12HSL) (verified in different settings with the used cancer cell lines)	[21]

↑: increased expression level; ↓: decreased expression level
